# Fast two-dimensional grid and transmission X-ray microscopy scanning methods for visualizing and characterizing protein crystals

**DOI:** 10.1107/S1600576716006233

**Published:** 2016-05-16

**Authors:** Justyna Aleksandra Wojdyla, Ezequiel Panepucci, Isabelle Martiel, Simon Ebner, Chia-Ying Huang, Martin Caffrey, Oliver Bunk, Meitian Wang

**Affiliations:** a Swiss Light Source, Paul Scherrer Institute, 5232 Villigen PSI, Switzerland; b Membrane Structural and Functional Biology Group, School of Medicine and School of Biochemistry and Immunology, Trinity College Dublin, Dublin 2, Ireland

**Keywords:** macromolecular crystallography, fast grid scans, oscillation images, scanning transmission X-ray microscopy, visualization of protein crystals

## Abstract

A fast continuous grid scan protocol has been incorporated into the Swiss Light Source (SLS) data acquisition and analysis software suite on the macromolecular crystallography (MX) beamlines. Its combination with fast readout single-photon counting hybrid pixel array detectors (PILATUS and EIGER) allows for diffraction-based identification of crystal diffraction hotspots and the location and centering of membrane protein microcrystals in the lipid cubic phase (LCP) in *in meso in situ* serial crystallography plates and silicon nitride supports. Diffraction-based continuous grid scans with both still and oscillation images are supported. Examples that include a grid scan of a large (50 nl) LCP bolus and analysis of the resulting diffraction images are presented. Scanning transmission X-ray microscopy (STXM) complements and benefits from fast grid scanning. STXM has been demonstrated at the SLS beamline X06SA for near-zero-dose detection of protein crystals mounted on different types of sample supports at room and cryogenic temperatures. Flash-cooled crystals in nylon loops were successfully identified in differential and integrated phase images. Crystals of just 10 µm thickness were visible in integrated phase images using data collected with the EIGER detector. STXM offers a truly low-dose method for locating crystals on solid supports prior to diffraction data collection at both synchrotron microfocusing and free-electron laser X-ray facilities.

## Abstract 2

This article reports the incorporation of a fast continuous grid scan with both still and oscillation images into the Swiss Light Source macromolecular crystallography beamlines and its application in visualization of protein crystals with scanning transmission X-ray microscopy.

## 1. Introduction   

The development of third-generation low-emittance X-ray sources and advances in X-ray optics have made single-digit micrometre-sized beams available for macromolecular crystallography (MX) at synchrotron beamlines worldwide (Smith *et al.*, 2012 ▸). Micrometre-sized X-ray beams have enabled MX with crystals as small as a few micrometres in their maximum dimension (Coulibaly *et al.*, 2007 ▸) and the location of small volumes in larger crystals with the best diffraction (Bowler *et al.*, 2010 ▸). The accurate centering of a micrometre-sized crystal in a micrometre-sized X-ray beam by visible-light microscopy, even with the aid of a high-resolution on-axis camera, is challenging owing to parallax, and borders on the impossible for many membrane protein crystals grown in and harvested from the lipid cubic phase (LCP) because of its frequent opacity at cryogenic temperatures (Caffrey *et al.*, 2012 ▸). In the latter case, the X-ray-diffraction-based grid scan (often referred to as rastering) proved to be indispensable for the structure determination of G protein-coupled receptors (Cherezov *et al.*, 2009 ▸). In a standard discrete grid scan a loop containing crystals is divided into a two-dimensional grid with a cell size matching that of the X-ray beam. A series of diffraction images (still or oscillation) are then taken from each cell as it is translated into the X-ray beam center in a discrete stepwise manner. Subsequently diffraction images are analyzed automatically (Zhang *et al.*, 2006 ▸) and the results represented graphically as a heat map for the rapid location of diffracting crystals. However, with typical loop sizes of 100–200 µm discrete grid scanning using a micrometre-sized X-ray beam is a time-consuming process. This becomes particularly burdensome when a large number of loops must be screened, as is the case in most structural biology projects. The introduction of the hybrid pixel array PILATUS 6M detector (Henrich *et al.*, 2009 ▸) has enabled continuous and shutterless data acquisition in MX. With a short readout time of 2.3 ms for the 6M detector (1 ms for the PILATUS3 model; http://www.dectris.com) it has been possible to implement two-dimensional continuous grid scanning with still diffraction images, where the sample is translated without rotation across the X-ray beam while the PILATUS detector acquires data continuously (Aishima *et al.*, 2010 ▸; Evans *et al.*, 2011 ▸; Svensson *et al.*, 2015 ▸). Two-dimensional continuous grid scanning is significantly faster than discrete stepwise scanning. However, the still diffraction images collected in this mode may provide less information than oscillation images.

Improvements in X-ray optics resulting in smaller beam sizes and higher intensity allow measurements on increasingly smaller protein crystals. As a consequence, radiation damage quickly limits data collection and impedes structure determination (Holton, 2009 ▸; Holton & Frankel, 2010 ▸). Even though diffraction-based grid scanning experiments lead to orders-of-magnitude lower accumulated dose than during the collection of a standard full dataset, there still exists a need for alternative low-dose imaging methods to localize crystals. The need is particularly apparent for (i) room-temperature data collection; (ii) micrometre-sized crystals with which only partial datasets can be recorded and where a single grid scan exposure represents a significant portion of the total tolerable radiation dose; (iii) the pre-location of crystals on solid supports for use in free-electron laser experiments. Second-order nonlinear imaging of chiral crystals (SONICC) is an optical method used for visualizing protein crystals in crystallization trays before mounting on the X-ray beamline (Kissick *et al.*, 2010 ▸) or in-line (Madden *et al.*, 2013 ▸). SONICC selectively detects noncentrosymmetric crystals. Unfortunately, it is not sensitive to crystals in certain space groups and it cannot discriminate between crystals of proteins and other chiral molecules that may be present in the crystallization drop (Haupert & Simpson, 2011 ▸). The application of X-ray microradiography and microtomography to the visualization and location of crystals in opaque media has been demonstrated previously (Brockhauser *et al.*, 2008 ▸; Warren *et al.*, 2013 ▸). However, X-ray microradiography requires a specialized experimental configuration, typically a large defocused X-ray beam in both vertical and horizontal directions to cover the entire sample and a high-resolution X-ray detector that includes a scintillator, a mirror on a translation stage and a CCD detector. Radiography only works when there is sufficient X-ray absorption contrast. This, in turn, requires delivery of a significant radiation dose to the sample. In contrast to this full-field imaging approach, scanning transmission X-ray microscopy (STXM) is a method in which an X-ray microbeam is scanned over the sample and the intensity of the transmitted beam is recorded as a function of sample position. Sophisticated detection schemes, such as differential phase contrast (DPC) and dark-field imaging, provide additional contrast modes and supplementary information about the sample. Importantly, they do not depend on delivering a high potentially damaging X-ray dose to the sample as they are based on refraction and elastic scattering (Bunk *et al.*, 2009 ▸; Thibault *et al.*, 2009 ▸; Menzel *et al.*, 2010 ▸).

In STXM experiments, typically a monochromatic X-ray beam is focused on the sample using a Fresnel zone plate with a pinhole blocking higher diffraction orders and a central spot blocking the unfocused beam. Data are collected by moving the sample stepwise through the X-ray beam and measuring the transmitted photon flux at each position in the area scanned (Bunk *et al.*, 2009 ▸; Thibault *et al.*, 2009 ▸). The transmission is obtained by integrating photon counts over the detector area on which the direct beam impinges. The image so obtained is in essence an X-ray radiograph, which amounts to a shadow image of the sample. As the X-rays pass through the sample they also undergo refraction. This changes the direction of the transmitted X-ray beam slightly, usually in the microradians range. With typically employed pixelated detectors and sample-to-detector distances, the shift in the position of the transmitted beam on the detector is well below the size of a detector pixel. Nevertheless, it can be reliably detected if several pixels are illuminated and the beam center is calculated, for example using the center of mass of the intensity pattern. The refraction signal recorded in this way yields DPC images in both horizontal and vertical directions. A key advantage of STXM is that it does not require additional optical elements in the beam path between sample and detector. It can be performed at a non-dedicated setup such as the Swiss Light Source (SLS) MX beamlines by simple beam conditioning and a repositioning of the detector and beamstop, procedures that can be easily automated.

Here, we describe a fast continuous diffraction-based two-dimensional grid scan method for use with and without sample oscillation. We also report on the use of the same scan method for low-dose detection of protein crystals with STXM. We show that STXM measurements on protein crystallography samples can be performed on the spot and provide useful crystal location information for subsequent efficient crystallographic data collection. This STXM application illustrates how the newly implemented grid scan methodology can serve as a versatile platform for a wide variety of X-ray scattering-based sample characterization methods. STXM is shown to work with crystals in nylon loops, on silicon nitride solid supports and in *in meso in situ* serial crystallography (IMISX) plates. These two-dimensional scanning methods not only dramatically reduce the time for screening and for diffraction-based alignment, but also provide a practical means for realizing high-throughput serial crystallography at both cryogenic and room temperature at synchrotron beamlines.

## 2. Methods   

### 2.1. Beamline setup   

**Figure 1:**
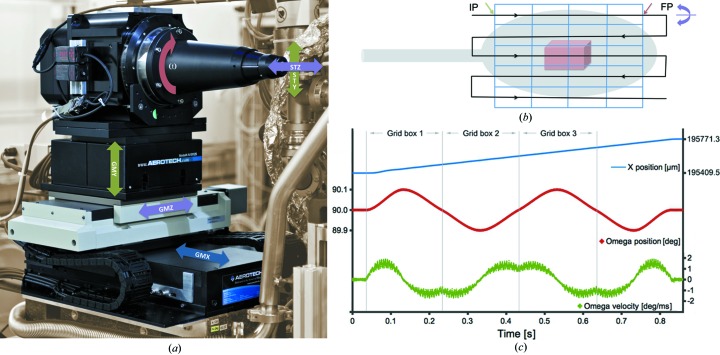
Scanning motions. (*a*) The D3 diffractometer motion stages with the corresponding axes labeled. (*b*) Schematic representation of a 4 × 5 grid. The initial (IP) and final (FP) positions within a scan line are labeled accordingly, as well as shutter open (green arrow) and shutter close (red arrow) positions. (*c*) Changes in omega angle and velocity over time during a camtable synchronized motion of GMX across a grid scan of three cells. The measurement was performed with an exposure time of 0.2 s and an oscillation of 0.2°. Data were monitored and collected with the Aerotech controller.

The MX beamline X06SA at the SLS uses a right-handed coordinate system with the *z* axis (GMZ in Fig. 1 ▸ *a*) along the X-ray beam direction, the *y* axis (GMY) pointing up and the ω rotation clockwise around the *x* axis (GMX). The D3 diffractometer (Fuchs *et al.*, 2014 ▸) implements a sample positioning system with a stack of three linear translation stages (Aerotech models ALS25020, ATS20010 and AVSI125 as GMX, GMY and GMZ, respectively; unidirectional repeatability of less than 1 µm) and an air-bearing spindle for the ω rotation (Aerotech ABRT 200; unidirectional repeatability of 0.5′′). A sample head consisting of two orthogonally mounted linear stages (SmarAct model SLC-1720-S as STY and STZ; 0.001 µm resolution), which support the sample magnet, is used to bring the region of interest of the sample onto rotation axis ω. Axes GMX, GMY, GMZ and ω are driven by an Aerotech A3200 controller supporting synchronized motions, while axes STY and STZ are driven by SLS standard motor drivers controlled *via* EPICS (Experimental Physics and Industrial Control System), which in the current configuration are not capable of synchronized motions. The focal spot size (FWHM) at beamline X06SA is 85 × 10 µm [horizontal (h) × vertical (v)] with the one-stage focusing mode and 10 × 1 µm (h × v) with the two-stage focusing mode (to be published). At a wavelength of 1 Å (12.4 keV) beamline X06SA delivers an X-ray flux of 2 × 10^12^ photons s^−1^. Diffraction images were collected with a PILATUS 6M-F (Henrich *et al.*, 2009 ▸) or an EIGER 16M (Dinapoli *et al.*, 2011 ▸; Tinti *et al.*, 2015 ▸) single-photon counting hybrid pixel area detector (Dectris Ltd, Baden, Switzerland).

### 2.2. Implementation of grid scan motions   

The grid scan protocol is available at all three MX beamlines at the SLS. During a scan, sample movement is performed and controlled with the D3 diffractometer, as described above (Fig. 1 ▸ *a*). Apart from a multi-line two-dimensional grid scan and single-line vertical and horizontal scans with still diffraction images, a unique feature of the system in place at the SLS is that it enables single- and multi-row grid scans to be performed synchronously with sample oscillation. The short readout time of the PILATUS and EIGER detectors provides continuous shutterless data acquisition while the sample is being translated along a row or column in the grid.

#### 2.2.1. Position synchronized triggering   

A position synchronized output circuit within the motion controller issues a transistor–transistor logic (TTL) signal within preprogrammed regions (start of a row/column) of the GMX or the GMY axis. The TTL signal is split and sent to both the shutter controller and the detector. The triggering of the detector used to record the diffraction images depends on whether a PILATUS or an EIGER detector is being used. The EIGER detector can be configured to collect *n* series, each consisting of *m* images (*i.e. n* rows by *m* columns), where each series is triggered by one external TTL signal. The PILATUS detector is configured to collect *m* × *n* images (*extmtrig* camserver command), each requiring an external trigger, which is delivered by a digital delay generator (Stanford Research Systems DG645). The DG645 is configured to replicate the incoming trigger from the motion controller into a sequence of *m* triggers along each row of the grid scan.

#### 2.2.2. Continuous grid scan with oscillation   

During the ‘grid scan with oscillation’ (Fig. 1 ▸ *b*) the GMX and ω axes are synchronized *via* a camtable with GMX as the master motor. The camtable is an array of *n* elements where each element is a pair of motor positions (GMX, ω) calculated to allow for ω oscillation simultaneously with the GMX movement along a row (the ω axis is parallel to the GMX axis; Fig. 1 ▸ *c*). The sequence of events required for such a scan is as follows: (i) the sample head is positioned so that the first cell of the grid is centered on the beam position and the first row is aligned along the ω rotation axis; (ii) the motion controller moves GMX to the first position of the camtable (IP in Fig. 1 ▸ *b*) and subsequently engages the GMX and the ω axes (from this point on all GMX motion will result in a synchronized ω motion); (iii) a linear move command is given on the GMX axis to reach the last position in the camtable (FP in Fig. 1 ▸ *b*), and this will effectively move the sample across the beam in order to scan the first row while the ω axis oscillates according to the camtable; (iv) after the movement is finished, the sample head moves the sample to align the next row on the ω rotation axis and the motion controller waits for a ‘start-next-row’ command; (v) the ‘start-next-row’ command is given by the data acquisition server to start another linear movement in the reverse direction towards the initial position. This procedure is repeated for as many rows as requested by the user.

#### 2.2.3. Continuous grid scan without oscillation   

In the ‘continuous grid scan without oscillation’ the procedure is the same as in the case of grid scan with oscillation (up to step iii) except for the camtable synchronization steps. The procedure continues as follows: (iv) instead of the sample head (as in ‘scan with oscillation’), the GMY axis moves the sample vertically to the next row with minimum delay; (v) the command for linear movement of GMX towards the initial position is issued. This procedure is repeated for as many rows as requested by the user. Similarly, for the vertical line scan without oscillation movements are accomplished with the GMY translation stage.

### 2.3. Computational setup   

The data acquisition and analysis software (*DA+*) includes components written in Python and Java which communicate *via* a messaging broker and deliver diffraction images through distributed messaging (to be published). The major components of *DA+* are the graphical user interface (GUI) based on the Java Eclipse rich client platform, which provides for user-friendly management of data collection with both standard and advanced protocols, the *DA+* server, which controls the workflow and interacts with the distributed real-time hardware control system EPICS, and the online analysis software for processing of grid scan images. *DA+* is deployed at all three MX beamlines (X06SA, X06DA, X10SA) at the SLS. Each MX beamline is equipped with a dedicated computing cluster enabling fast processing of data stored on a GPFS file server. For the computation-intensive diffraction-based grid scan experiments an additional cluster (referred to as the raster cluster) has been installed, consisting of three 24-core nodes, each with 2.70 GHz CPUs and 256 GB RAM.

### 2.4. Processing of grid scan data   

When a grid scan data collection session is started, multiple processes running in the raster cluster receive compressed images collected by the detector as ZeroMQ messages (*via* PUSH/PULL; http://zeromq.org), decompress the messages and immediately analyze them, whilst in memory, using routines from the *labelit.distl* package (Zhang *et al.*, 2006 ▸). Results are reported back to the *DA+* GUI *via* the messaging broker and are displayed in the GUI. *Albula* software (Dectris) displays selected diffraction images during data collection and enables interactive inspection of specific diffraction images based on the results of the evaluation.

### 2.5. Grid scan in the *DA+* GUI   

**Figure 2:**
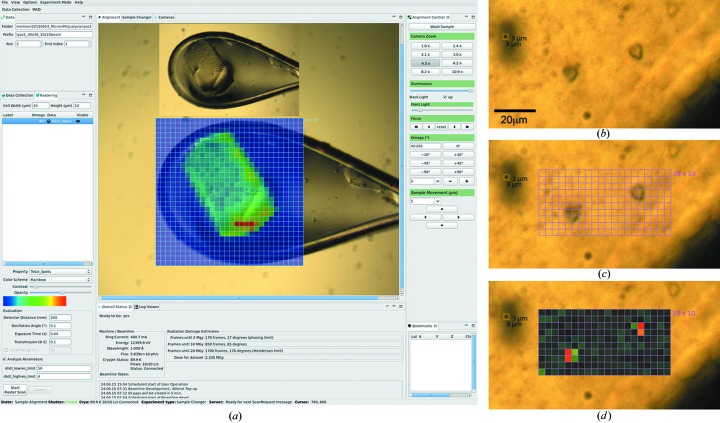
Implementation of the fast grid scan in the *DA+* GUI. (*a*) In the sample camera view the user can define the grid scan area and data collection parameters. Collected diffraction images are analyzed with *DISTL* and displayed with the color scheme of choice in the sample camera window. Analysis criteria such as property and resolution limits can be modified. The inset shows a photograph of the crystal in the loop. (*b*)–(*d*) Localization of microcrystals in the LCP on silicon nitride support at 100 K with the 3 × 3 µm microbeam. (*b*) On-axis microscope image. (*c*) Definition of the diffraction-based grid scan with 3 × 3 µm cells in the user interface. (*d*) Heat map of the diffraction results with red corresponding to the strongest diffraction.

The data collection window in the *DA+* GUI includes a rastering tab where data acquisition parameters such as detector distance, oscillation angle, exposure time and X-ray transmission are defined (Fig. 2 ▸ *a*). A rectangular grid scan area is defined and overlaid on the in-line camera view of the sample by mouse click-and-drag procedures. In the standard setup, the grid cell width and height correspond to those of the beam. During data collection, images are displayed in the *Albula Viewer* and saved in a location with a unique folder name specified by the user. By default, images are ranked on the basis of the number of Bragg peak candidates in each grid cell as evaluated by *DISTL* and are displayed in the camera view as a heat map superimposed on the original image. The ranking property can be changed in the drop-down menu, as can other analysis parameters, such as high- and low-resolution limits. Moreover, the user can modify the color scheme, contrast and opacity of the displayed heat map.

### 2.6. Grid scan experiments   

Grid scan experiments were carried out with the two-stage focusing setup at beamline X06SA using a PILATUS 6M-F detector. To obtain a horizontal beam size smaller than 10 µm at the sample position, the beam was reduced with slits at the position of the intermediate focus. In the vertical direction a beam larger than 2 µm at the sample position was achieved by moving the focal point downstream with the vertically focusing mirror. A 10 × 10 µm beam with a flux of 5.8 × 10^10^ and 8.2 × 10^11^ photons s^−1^ was used in the experiments shown in Figs. 2 ▸(*a*) and 3 ▸, respectively. A 3 × 3 µm beam with a flux of 8 × 10^10^ photons s^−1^ was used in the experiment shown in Fig. 2 ▸(*b*).

### 2.7. STXM   

For the STXM experiment, conducted at 12.4 keV on beamline X06SA with the one-stage focusing setup, the X-ray beam was defocussed in both the horizontal and vertical directions. Subsequently, the beam size was reduced with slits to 10 × 10 µm (experiment shown in Fig. 4 ▸). In the case of the two-stage focusing setup, the beam was focused on the sample to the desired size of 10 × 10, 3 × 6 or 3 × 3 µm (experiments shown in Fig. 5 ▸). Both approaches yielded a uniform, well defined beam profile suitable for STXM experiments. To enable measurement of the transmitted intensity at each grid point the beamstop was moved out of the direct X-ray beam. The detector (either PILATUS 6M-F or EIGER 16M) was moved to a distance of 1200 mm from the sample to achieve high sensitivity to changes in the direction of the X-ray beam due to its refraction by the sample. The measurements were performed at very low incident intensity below a global count rate of 6 × 10^6^ photons per second and per pixel for the transmitted beam, which corresponds to about 300 Gy s^−1^ for a 3 × 3 µm beam (Holton, 2009 ▸). STXM data were processed with in-house MATLAB (The MathWorks Inc., Natick, MA, USA) routines to obtain transmission and DPC images of the measured samples (Bunk *et al.*, 2009 ▸).

### 2.8. Crystals   

**Figure 3:**
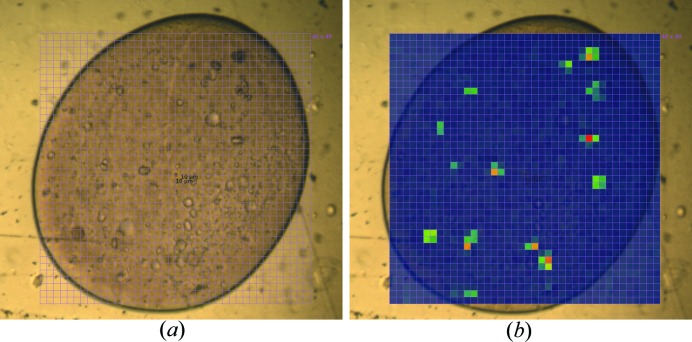
A diffraction-based grid scan covering a whole LCP bolus allows diffraction-based identification of crystals suitable for further small-wedge data collection. Images show a 50 nl bolus containing crystals of PepT_St_ in an IMISX COC-windowed well. Data were collected at 100 K with a PILATUS 6M-F detector. (*a*) Definition of the grid scan area with 400 × 400 µm cells. (*b*) Heat map of the diffraction intensity detected in the grid scan.

Insulin crystals were obtained in a cryo-protective condition consisting of 25–32% *v*/*v* ethylene glycol and were harvested with 20 µm diameter nylon loops (Hampton Research; Figs. 2 ▸ *a* and 4 ▸). Chicken egg-white lysozyme was crystallized *in meso* in IMISX plates using 200 nl mesophase and 1000 nl precipitant solution, as described previously (Huang *et al.*, 2015 ▸, 2016 ▸). After removal from the outer glass sandwich plate, part of the inner cyclic olefin copolymer (COC)-windowed sandwich plate with eight crystallization wells was mounted on the goniometer (Fig. 5 ▸ *b*). Glucose isomerase crystals were obtained in a crystallization condition that included 0.1 *M* sodium cacodylate pH 6.5, 0.2 *M* magnesium acetate and 30%(*v*/*v*) 2-methyl-2,4-pentanediol. Silicon nitride (Si_3_N_4_) solid supports (1.5 × 1.5 mm, 1 µm-thick silicon nitride membrane window centered within a 5 × 5 mm, 200 µm-thick silicon frame; Silson Ltd, Northhampton, England) were glued with epoxy adhesive to a loopless pin mounted on a magnetic cryocap (Molecular Dimensions). One microlitre of crystallization mother liquor containing crystals of glucose isomerase was placed on the window of the silicon nitride solid support and immediately snap-cooled in liquid nitrogen for use in data collection (Fig. 5 ▸ *a*). PepT_St_ (peptide transporter from *Streptococcus thermophilus*) was crystallized by the *in meso* method using either 50 nl (IMISX plate; Fig. 3 ▸) or 150 nl (silicon nitride plate; Fig. 2 ▸ *b*) of mesophase and 1000 nl of precipitant solution (Huang *et al.*, 2015 ▸). Crystallization boluses containing crystals were cut out from the crystallization plate and mounted onto a magnetic cryocap for data collection at 100 K, as described previously (Huang *et al.*, 2016 ▸).

## 3. Results and discussion   

### 3.1. Diffraction-based grid scan   

In the first example of a diffraction-based grid scan experiment a large ∼200 × 100 µm insulin crystal was snap-cooled to 100 K in a standard nylon loop (Fig. 2 ▸ *a* inset). A fast grid scan with oscillation was recorded at 12.4 keV at beamline X06SA in the two-stage focusing mode. The beam size was adjusted to 10 × 10 µm, which gave a total flux of 5.8 × 10^10^ photons s^−1^ at 10% transmission. Data collection was performed with a PILATUS 6M-F detector at 25 Hz (oscillation angle, 0.1°; exposure time, 40 ms) and a detector distance of 200 mm. With a 30 × 30 grid corresponding to an area of 300 × 300 µm, all 900 images were collected in 36 s. The diffraction images were processed on dedicated computing nodes and the results were displayed almost immediately after data collection (Fig. 2 ▸ *a*). The results of an analysis performed with the *DISTL* program using the total-number-of-spots option and a resolution range from 50 to 4 Å are shown as a heat map in Fig. 2 ▸(*a*). The shape of the crystal can be clearly seen, as can the diffraction ‘hotspot’ in the crystal, which in this case corresponds to the thickest part of the crystal.

Diffraction-based grid scans are commonly used with a microbeam to identify microcrystals for micro- and serial crystallography of challenging targets such as membrane proteins (Smith *et al.*, 2012 ▸; Huang *et al.*, 2015 ▸). In the example shown in Fig. 2 ▸(*b*), crystals of the membrane protein PepT_St_ in LCP between silicon nitride windows were located with a grid scan. Data were collected with a 3 × 3 µm beam, delivering a flux of 8 × 10^10^ photons s^−1^ at 50% transmission using the PILATUS 6M-F detector at 10 Hz (oscillation angle, 0.1 °; exposure time, 0.1 s) and a detector distance of 400 mm. It took 20 s to collect and analyze the 200 diffraction images recorded over the 20 × 10 grid, having an area of 60 × 30 µm. Bragg spot evaluation allowed for the accurate location of a small (6 × 6 µm) PepT_St_ crystal with the 3 × 3 µm microbeam. The example shown in Fig. 2 ▸(*b*) demonstrates that microcrystals grown in the LCP can be identified readily with a diffraction-based grid scan. However, the region of interest examined in this case is relatively small. Often when working with LCP samples a large volume of mesophase containing tiny crystals must be interrogated. Fig. 3 ▸ shows a large 50 nl mesophase bolus containing microcrystals of PepT_St_ in an IMISX plate. It was scanned with a 10 × 10 µm beam (8.2 × 10^11^ photons s^−1^) over a 40 × 40 grid covering an area of 400 × 400 µm. All 1600 images were recorded in 64 s with the PILATUS 6M-F detector operating at 25 Hz (oscillation angle, 0.4°; exposure time, 40 ms). The recent installation of the EIGER 16M detector on beamline X06SA can reduce the time needed to collect the same number of images to just 12 s when operating at 133 Hz. Assuming sufficient flux, the pronounced reduction in diffraction-based grid scan data collection time will dramatically improve the efficiency and throughput of serial crystallography at synchrotrons. Typically this will involve an initial ultra-rapid grid scan to identify well diffracting crystals in the mesophase followed by standard data collection where small wedges of diffraction data are acquired on selected crystals in the bolus.

### 3.2. STXM   

STXM measurements were performed at beamline X06SA at the SLS with the experimental setup described in §[Sec sec2.7]2.7. Data were collected at an energy of 12.4 keV and at the very low incident beam intensity of ∼10^6^ photons s^−1^, which is 4–5 orders of magnitude lower than the typical flux (∼10^10^–10^11^ photons s^−1^) used for collecting diffraction data. Apart from reducing the experimental overhead for STXM measurements by avoiding changes of the X-ray energy, collecting data at this commonly used energy provides additional advantages in terms of imaging at low deposited X-ray doses, as explained in the following. According to the literature, protein crystal density values range from 1.22 to 1.47 g cm^−3^ with an average of 1.35 g cm^−3^ (Matthews, 1968 ▸; Andersson & Hovmöller, 1998 ▸; Quillin & Matthews, 2000 ▸; White *et al.*, 2007 ▸). By contrast, the density of the crystallization media used in the current study is very close to 1.00 g cm^−3^, the highest being 1.09 g cm^−3^ for the lysozyme crystallization condition (calculated with the online calculator http://sednterp.unh.edu). The dependence of the real and imaginary parts of the refractive index of protein crystals and crystallization solutions on the X-ray energy has two important properties worth considering in light of our STXM measurements. The absorption contrast given by the imaginary part of the refractive index is much higher at 6 keV than at 12.4 keV. However, maximizing absorption is a disadvantage because it also maximizes radiation damage directly linked to it. On the other hand, at 12.4 keV the difference in the real part of the refractive index of crystal and solution is relatively pronounced (it decays less as a function of energy than the imaginary part). This gives rise to a more sensitive DPC signal in comparison with the absorption (transmission) signal at these hard X-ray energies.

**Figure 4:**
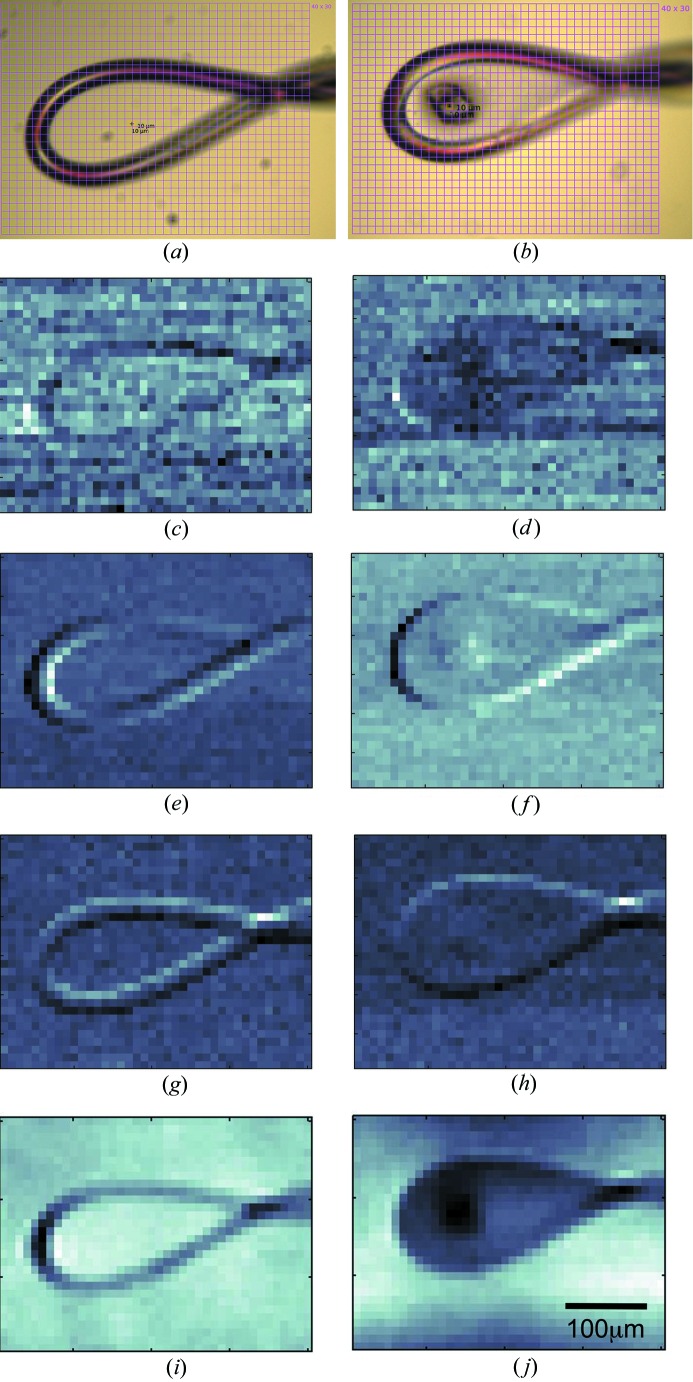
Loop and crystal detection with STXM. Panels on the left show data obtained for an empty nylon loop. Panels on the right show the same loop with a flash-frozen 40 × 40 µm insulin crystal. Data were collected at 100 K with a PILATUS 6M-F detector. (*a*), (*b*) On-axis light microscope image, (*c*), (*d*) X-ray transmission, (*e*), (*f*) differential phase contrast in the **x** direction, (*g*), (*h*) differential phase contrast in the **y** direction, (*i*), (*j*) integrated phase. The crystal is clearly visible in the integrated phase image.

Fig. 4 ▸ shows the STXM results obtained for an empty nylon loop and the same loop containing a flash-frozen insulin crystal. Data were collected using a 10 × 10 µm beam and a fast grid scan with a grid size of 40 × 30 and 0.1 s exposure. In total 1200 images were collected in 120 s. The size and shape of the loop are clearly discernible in the transmission image, as well as in the differential phase contrast and integrated phase images. The crystal, which is approximately 40 × 40 µm in size, can be clearly identified in all images. Upon close inspection of the data in Fig. 4 ▸, lines of dark spots or dots at certain locations in the absorption images can be seen. These can be accounted for by recognizing that the SLS ring operates in top-up mode with a stored current of 400 ± 1 mA, which is maintained by injections every 1–2 min. The top-up injection process transiently increases the diameter of the electron beam. As seen through the beam-defining slits of an MX beamline, a slight and transient drop in beam intensity occurs. The data included in this study have not been normalized with respect to incident photon flux. Hence, the regular small reductions in the intensity of the transmission images observed during the course of data collection reflect these periodic injections.

**Figure 5:**
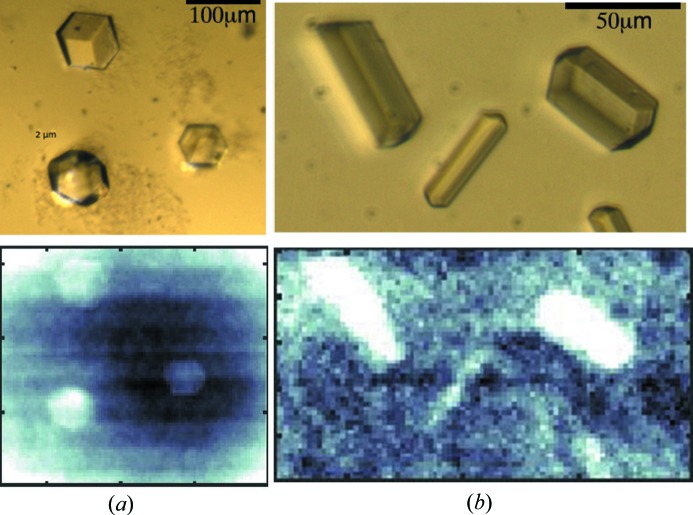
Localization of crystals in integrated phase images obtained with STXM. (*a*) Glucose isomerase crystals, 50 × 50 µm in size, deposited onto a silicon nitride solid support are clearly visible. Data were collected at 100 K with the PILATUS 6M-F detector. (*b*) Lysozyme crystals with a diameter of 10 µm are detectable. Crystals were grown *in meso* in an IMISX plate. Data were collected at room temperature with the EIGER 16M detector. The top panels show light microscope images, while the bottom panels show integrated phase images. The phase images are displayed on an inverted gray scale in comparison to Fig. 4 ▸(*i*) and 4 ▸(*j*) to improve visibility.

X-ray free-electron lasers (FELs) provide high-intensity pulses of femtosecond duration, enabling the collection of high-resolution diffraction patterns from protein crystals before complete destruction by radiation damage (‘diffraction before destruction’) (Schlichting, 2015 ▸). Only a single still diffraction image can be collected from each femtosecond exposure, and the sample must be renewed between each shot. The macromolecular micro- or nanocrystals used in serial femtosecond crystallography (SFX) can be delivered into the FEL beam using a variety of jet devices (Weierstall, 2014 ▸) or mounted on a solid support (fixed target or chip) (Hunter *et al.*, 2014 ▸; Feld *et al.*, 2015 ▸). In such delivery systems, crystal orientation is ideally random and it is necessary to collect patterns on thousands of crystals to produce a complete dataset of still images. However, not every FEL pulse hits a crystal or generates a useful diffraction pattern. An approach to optimize hit rates involves establishing the location of crystals on the solid support prior to the SFX study. It is in such an application that STXM may prove useful and tests to evaluate this were performed in the current study. Measurements with a 3 × 3 µm beam were carried out with 50 × 50 µm-sized glucose isomerase crystals deposited onto a silicon nitride support, using a PILATUS 6M-F detector operating at 20 Hz. The data in Fig. 5 ▸(*a*) show that, despite the limited resolution, the method clearly works. We believe that STXM performed prior to the FEL experiment on a synchrotron beamline optimized for such measurements will enable the successful location of microcrystals on solid supports and, in turn, faster and more efficient SFX data collection.

The maximum resolution provided in an STXM measurement is limited by the size of the beam on the sample (Thibault *et al.*, 2008 ▸). The recent X06SA beamline optics upgrade enables measurements with a beam size down to 2 × 1 µm. Despite this diminutive beam size, currently a crystal size of 40 × 40 µm appears to be the lower limit for the detection of protein crystals by STXM on a solid support and in a nylon loop at the X06SA MX beamline. This may be because, despite mimicking a standard STXM experiment as closely as possible, the experimental configuration used in the current study was not ideal in two important ways compared to what is available at dedicated imaging beamlines. First, vibrations that cause the beam position to fluctuate slightly affect a crystallography experiment much less than an imaging experiment, although as smaller and smaller beam sizes are used in MX, beam stability requirements continue to rise. Relatedly, imaging measurements are used to quantify beam stability. The second important factor is contrast related to beam conditioning and the detector. Even though the X-ray beam is focused on the sample, the beam divergence is much smaller than prevails at a typical imaging station. Thus, the size of the beam on the detector, even though the detector is 1200 mm away from the sample in this study, is such that the beam only strikes a very few central pixels. This, in turn, impairs the beam position determination and subsequent angular beam-deflection measurement resolution. To investigate this further we performed STXM experiments with an EIGER detector. The EIGER is a hybrid photon counting pixel area detector with a pixel size of 75 × 75 µm (*cf.* 172 × 172 µm in the case of PILATUS) equipped with a 12 bit single-photon counter. The combination of frame rates of about 750 Hz, flexibility to define various sizes of the region of interest (depending on the detector model and operation mode) and continuous readout with a dead time of only 3.8 µs between exposures makes it ideally suited to ultra-fast grid scan experiments. STXM data were collected at room temperature over a 90 × 50 grid at 50 Hz corresponding to 4500 images acquired in 90 s with a beam size of 3 × 6 µm. Microcrystals of lysozyme grown in LCP were clearly visible in the integrated phase image (Fig. 5 ▸ *b*). Using the EIGER 16M detector, with a pixel size about five times smaller in area than that of the PILATUS detector, thin needles with a thickness of 10 µm were successfully identified.

## 4. Summary and conclusions   

Rastering of samples has been incorporated as a standard technique easily accessible *via* a GUI at many synchrotron MX beamlines around the world (Song *et al.*, 2007 ▸; Cherezov *et al.*, 2009 ▸; Aishima *et al.*, 2010 ▸; Bowler *et al.*, 2010 ▸; Hilgart *et al.*, 2011 ▸). Diffraction-based grid scans are particularly useful in locating small crystals in opaque media such as the LCP, in identifying diffraction ‘hotspots’ in crystals and, in combination with a small intense beam, in serial X-ray crystallography. Fast diffraction-based alignment is more efficient than the traditional manual crystal centering with an in-line light microscope and other techniques such as tomography or SONICC, in the sense that these do not provide information about diffraction quality of crystals. In this paper, we present to our knowledge one of the fastest continuous two-dimensional grid scanning protocols that includes collection of still images. The experimental setup at the SLS offers the added unique feature of being able to collect oscillation images during the fast and continuous grid scanning process. While no systematic advantage of collecting oscillation images could be clearly identified for all types of samples, we believe it is a complementary approach to still image data collection that helps in dealing with difficult samples on a case-by-case basis. In our experience the decision concerning grid scan data collection in the static or oscillation mode is very much crystal dependent. For crystals with high mosaicity it is advisable to perform the grid scan with still images, while in the case of crystals with low mosaicity a grid scan with oscillation images is preferred. We plan to implement an automatic loop localization coupled with crystal centering based on grid scan diffraction to enable fully automated screening and data collection at our MX beamlines. The installation of an EIGER 16M detector at the X06SA beamline, which can achieve frame rates of 133 Hz for the full frame and 750 Hz when the central 4M pixels are read out as the region of interest, will significantly further improve data collection speed.

The combination of straightforward experimental setup and ultra-fast grid scanning has been shown to be effective in low-dose detection of protein crystals by STXM. With this method, crystals in standard nylon loops, on solid supports and in *in situ* plates were successfully identified and localized, at both cryogenic and room temperatures. The results presented in this paper are a proof of principle that STXM is an effective method for localizing macromolecular crystals directly at MX beamlines. They are particularly relevant in light of the growing popularity of FEL SFX with micrometre-size crystals of challenging targets such as membrane proteins. The accurate and precise location of crystals on a solid support *via* low-dose X-ray imaging at a synchrotron or a laboratory source prior to measurements will facilitate fast and efficient SFX data collection. Moreover, at MX beamlines phase-contrast imaging, for example employed in a STXM experiment, provides a much lower dose alternative to the standard diffraction-based methods for locating crystals in opaque media. The experimental setup available at an MX beamline could be used for performing such experiments prior to crystallographic data collection.
